# Clinical Implications of Plasma Epstein–Barr Virus DNA in Children and Adolescent Nasopharyngeal Carcinoma Patients Receiving Intensity-Modulated Radiotherapy

**DOI:** 10.3389/fonc.2020.00356

**Published:** 2020-03-31

**Authors:** Wenze Qiu, Xing Lv, Xiang Guo, Yawei Yuan

**Affiliations:** ^1^Department of Radiation Oncology, Affiliated Cancer Hospital & Institute of Guangzhou Medical University, Guangzhou, China; ^2^State Key Laboratory of Oncology in South China, Collaborative Innovation Center of Cancer Medicine, Sun Yat-sen University Cancer Center, Guangzhou, China

**Keywords:** nasopharyngeal carcinoma, children and adolescent, Epstein–Barr virus, intensity-modulated radiotherapy, clinical implications

## Abstract

**Background:** Plasma Epstein–Barr virus (EBV) DNA has been determined as a prognostic factor in adult nasopharyngeal carcinoma (NPC) patients. This study was designed to evaluate the prognostic value of plasma pretreatment EBV DNA in children and adolescent NPC patients receiving intensity-modulated radiotherapy (IMRT).

**Methods:** Pretreatment EBV DNA was retrospectively assessed in 147 children with newly diagnosed, non-metastatic NPC. All patients were treated using IMRT. Receiver operating characteristic (ROC) curve was used to identify the optimal EBV DNA cutoff point. Prognostic value was examined using a multivariate Cox proportional hazards model.

**Results:** The median follow-up for the entire cohort was 58 months (range, 10–119 months), and the 5-year survival rates for all patients were as follows: overall survival (OS), 88.7%; locoregional relapse-free survival, 95.2%; distant metastasis-free survival (DMFS), 84.8%; and disease-free survival (DFS), 81.5%. For ROC curve analysis, the optimal cutoff value of pretreatment EBV DNA load for DFS was 40,000 copies/mL. High plasma EBV DNA was significantly associated with poorer 5-year DMFS (70.6 vs. 89.1%, *P* = 0.003) and DFS (63.9 vs. 86.9%, *P* < 0.001). In multivariate analysis, high plasma EBV DNA was an independent predictor for DMFS and DFS.

**Conclusions:** Pretreatment EBV DNA level was a powerful prognostic discriminator for DMFS and DFS in children and adolescent NPC patients treated with IMRT.

## Introduction

Children and adolescent nasopharyngeal carcinoma (NPC) is rare, accounting for ~1% of all cases of NPC in the endemic areas of southern China (1). Although the incidence varies extensively with racial and geographical factors, it constitutes 1–5% of all malignant tumors and 20–50% of all primary nasopharyngeal malignant tumors in this age group (2–4). Children and adolescent NPC is distinguishable from the adult form of the disease because of its undifferentiated histology and the high incidence of advanced stage disease and its close association with Epstein–Barr virus (EBV) infection (1).

Because of the rarity of NPC in children, its epithelial cell origin, and occurrence in older children and adolescents, the treatment recommendations for childhood NPC typically follow guidelines established for adults. Radiotherapy (RT) is the primary treatment modality, and concurrent chemoradiotherapy with or without neoadjuvant/adjuvant chemotherapy is regarded as the standard of care for patients with locoregionally advanced NPC. In recent years, intensity-modulated radiation therapy (IMRT), which is associated with superior disease control and a lower treatment toxicity profile, has gradually replaced two-dimensional conventional radiotherapy as the mainstay RT technique for children and adolescent NPC patients (5).

Several clinical features, including age, gender, stage, RT technique, RT dose, and response to chemotherapy can predict the prognosis in children with NPC (5–10). However, the variable outcomes of patients within heterogeneous subgroups suggest that clinical features alone cannot precisely predict the treatment outcome. This prompted us to determine and evaluate prognostic factors tailored to children and adolescent patients.

Plasma EBV DNA is one of the most well-recognized biomarkers for NPC (11, 12). An expanding body of data has suggested that the EBV DNA load correlates with clinical stage and can be used for monitoring and prediction of the survival of NPC patients (13, 14). To the best of our knowledge, studies that explore whether treatment outcomes can be predicted by pretreatment levels of plasma EBV DNA in young NPC patients are rare. Although one retrospective study (15) has reported that plasma EBV DNA predicts worse outcomes in pediatric non-metastatic NPC patients, the findings were based on a relatively small sample size without using uniform IMRT technique.

Therefore, this retrospective study was conducted to confirm whether pretreatment plasma EBV DNA levels are able to accurately predict the prognosis of a large population of NPC patients in childhood and adolescence undergoing modern RT treatment.

## Materials and Methods

### Patient and Staging Evaluation

A total of 147 children with NPC were treated by IMRT at the Sun Yat-sen University Cancer Center from June 2008 to December 2015. Patients were 7 to 20 years of age and histologically diagnosed with untreated non-metastatic NPC. All patients underwent a pretreatment evaluation including a complete physical examination, magnetic resonance imaging (MRI)/computed tomography (CT) of the nasopharynx and neck, chest radiography, abdominal ultrasonography, and single-photon emission computed tomography whole-body bone scan. Positron emission tomography was optional and was performed when clinically indicated. Patients were restaged by two radiation oncologists specializing in head and neck cancer according to the eighth edition of the American Joint Committee for Cancer Staging system (16), with disagreements resolved by consensus. This retrospective study was conducted in compliance with the institutional policy to protect the patients' private information and was approved by the institutional ethical committee. Informed consent was obtained from the subject and/or guardian.

### Quantification of Plasma EBV DNA

Before the start of treatment, peripheral venous blood (3 mL) was collected from each patient into EDTA-containing tubes and centrifuged at 3,000 g for 5 min. Total plasma DNA was extracted using a QIAamp DNA Blood Mini Kit (Qiagen, Hilden, Germany). Fluorescence polymerase chain reaction (PCR) was carried out using an EBV PCR quantitative diagnostic kit (Da-An Genetic Diagnostic Center, Guangzhou, China) targeting the BamHI-W region of the EBV genome. Data were analyzed using Applied Biosystems 7300 SDS software (Beijing, China).

### Radiotherapy

All patients received IMRT as a primary treatment. The techniques of planning and delivery of IMRT were described previously (17, 18). Gross tumor volume (GTV) included the primary tumor and the enlarged lymph nodes. GTVnx included the sum of the primary tumor volume and the enlarged retropharyngeal nodes, whereas GTVnd was the volume of clinically involved gross cervical lymph nodes. High-risk clinical target volume (CTV1) was defined as the nasopharynx GTV plus a 5–10-mm margin (2–3 mm posteriorly if adjacent to the brainstem or spinal cord) to encompass the high-risk sites of the microscopic extension and the whole nasopharynx. Low-risk clinical target volume (CTV2) was defined as the high-risk clinical target volume plus a 5- to 10-mm margin (2–3 mm posteriorly if adjacent to the brainstem or spinal cord) to encompass the low-risk sites of the microscopic extension, including the skull base, clivus, sphenoid sinus, parapharyngeal space, pterygoid fossae, posterior parts of the nasal cavity, pterygopalatine fossae, retropharyngeal nodal regions, and the elective neck area from level IB to level V. A planning target volume (PTV) was created by adding a three-dimensional margin of 3–5 mm to the delineated target volume to compensate for the uncertainties in treatment setup and internal organ motion. The prescribed doses were 66–70, 64–70, 60–62, and 54–56 Gy, in 30–33 fractions, for the PTVs derived from GTVnx, GTVnd, CTV1, and CTV2, respectively. The dose constraints for organs at risk and planning organ at risk volumes were as described for the RTOG-0225 trial (19). All patients were treated following a routine schedule (one fraction daily, 5 days per week).

### Chemotherapy

Chemotherapy regimens and administering schedules had some heterogeneity. Whereas, three patients (2.0%) were treated by RT alone, 20 patients (13.6%) received neoadjuvant chemotherapy before RT, 34 patients (23.1%) received concurrent chemotherapy, and 90 patients (61.2%) received neoadjuvant and concurrent chemotherapy.

Neoadjuvant chemotherapy included the following regimens: PF [consisting of cisplatin (1 day of 80–100 mg/m^2^) and 5-fluorouracil (800–1,000 mg/m^2^, by 120-h continuous intravenous infusion)], TP [consisting of docetaxel (1 day of 75 mg/m^2^) or paclitaxel (1 day of 150–180 mg/m^2^) and cisplatin (1 day of 75 mg/m^2^)], and TPF [consisting of docetaxel (1 day of 60 mg/m^2^) or paclitaxel (1 day of 135 mg/m^2^), cisplatin (1 day of 60 mg/m^2^), and 5-fluorouracil (500–800 mg/m^2^, by 120-h continuous intravenous infusion)]. All regimens were administered at intervals of 3 weeks for two to four cycles. Concurrent chemotherapy consisted of cisplatin (80–100 mg/m^2^) given in weeks 1, 4, and 7 of RT, or cisplatin (30–40 mg/m^2^) given weekly during RT, beginning on the first day of RT.

### Evaluation Criteria and Follow-Up

The treatment outcome was evaluated according to the Response Evaluation Criteria in Solid Tumors (version 1.1). All patients were evaluated weekly during radiation therapy, with a required follow-up after they completed RT: every 3 months in the first 2 years, every 6 months from the third to fifth years, and annually thereafter. The following examinations should be included in the follow-up: physical examination, routine blood test, biochemistry, plasma EBV DNA test, nasopharyngoscopy, chest X-ray or CT scan, abdominal ultrasonography, and nasopharynx + neck MRI scan with contrast.

### Statistical Analysis

The following endpoints were assessed: locoregional relapse-free survival (LRRFS), distant metastasis-free survival (DMFS), disease-free survival (DFS), and overall survival (OS), and DFS was set as the primary endpoint. Locoregional relapse-free survival was measured from the end of RT to the date of the first observation of local or regional recurrence. Distant metastasis-free survival was measured from the end of RT to the date of the first observation of distant metastasis. Disease-free survival was measured from the end of RT to the date of the first observation of local or regional recurrence or distant metastasis. Overall survival was measured from the end of RT to the time of death or the time of last follow-up. We used χ^2^ test for categorical variables and Mann-Whitney *U* tests for continuous variables to assess the differences between groups. Receiver operating characteristic (ROC) curve was obtained by plotting sensitivity against 1–specificity to evaluate performance of EBV DNA for predicting DFS. The optimal cutoff point of EBV DNA was identified based on Youden index, which was at the maximum sum of the sensitivity and specificity−1. The area under the curve (AUC) was used to assess the prognostic value of EBV DNA. An AUC of 0.5 represents a test with no discriminating ability (i.e., no better than chance), whereas an AUC of 1.0 represents a test with perfect discrimination (20–24). The Kaplan–Meier method was used to calculate actuarial survival rates and to draw survival curves, and the differences were compared using the log-rank test. Multivariate analysis was performed using the Cox proportional hazards model to define the independent risk factors for survival rates. The Statistical Package for the Social Sciences software package (SPSS 25.0, SPSS, Inc., Chicago, IL) was used, and a two-tailed *P* < 0.05 was considered to be statistically significant.

## Results

### Patient Characteristics

Table 1 shows the demographic information of all the patients included in the study. The median age at diagnosis was 17 years (range, 7–20 years) with a male-to-female ratio of 2.97:1. Based on the World Health Organization (WHO) criteria, 98.0% of patients had type III disease, and 2.0% had type II disease. By TNM stage, 55 (37.4%) patients were at stage III, and 86 (58.5%) at stage IV. Chemotherapy was administered to 144 patients, whereas the other three were given RT alone. The median cumulative cisplatin dose during the entire treatment was 320 mg/m^2^ (range, 0–480 mg/m^2^).

**Table 1:** Clinical characteristics of 147 patients with children and adolescent nasopharyngeal carcinoma.

**Characteristics**	**No. of patients**	**%**
Age, years		
≤17	79	53.7
>17	68	46.3
Gender		
Male	110	74.8
Female	37	25.2
Pathologic type		
WHO II	3	2.0
WHO III	144	98.0
Pretreatment BMI, kg/m^2^		
<23	128	87.1
≥23	19	12.9
T Stage		
T1	4	2.7
T2	10	6.8
T3	61	41.5
T4	72	49.0
N Stage		
N0	4	2.7
N1	38	25.9
N2	77	52.4
N3	28	19.0
Overall stage		
I	1	0.7
II	5	3.4
III	55	37.4
IV	86	58.5
Combination with Chemotherapy		
No	3	2.0
NAC	20	13.6
CCT	34	23.1
NAC + CCT	90	61.2
Total dose of cisplatin, mg/m^2^ [median (range)]	320 (0–480)	
Pretreatment plasma EBV Dna		
0	26	17.7
<10^3^	14	9.5
<10^4^	37	25.2
<10^5^	50	34.0
<10^6^	19	12.9
<10^7^	1	0.7
Median level, copies/mL (interquartile range)	7,360 (660–39,200)	

*BMI, body mass index; NAC, neoadjuvant chemotherapy; CCT, concurrent chemotherapy*.

The median concentration of plasma EBV DNA in our study was 7,360 copies/mL (interquartile range, 660–39,200 copies/mL). The median pretreatment plasma EBV DNA levels were described as stratified by different classifications. Advanced T stage, N stage, and clinical stage had higher median pretreatment plasma EBV DNA levels; however, the differences did not reach statistical significance (all *P* > 0.05; Table 2).

**Table 2:** Associations between pretreatment EBV DNA and TNM staging.

**Characteristics**	**No. of patients**	**%**	**Median (copies/mL)**	**Interquartile range (copies/mL)**	***P***
T stage					0.743
T1–T3	75	51.0	5,790	110–41,800	
T4	72	49.0	9,890	900–35,350	
N stage					0.216
N0–1	42	28.6	4,325	60–40,825	
N2–3	105	71.4	10,500	1,915–39,500	
Overall stage					0.101
I–III	61	41.5	4,790	90–27,400	
IV	86	58.5	15,200	1,500–40,375	

### Correlations Between Patient Characteristics and Pretreatment Plasma EBV DNA Levels

**Figure 1:**
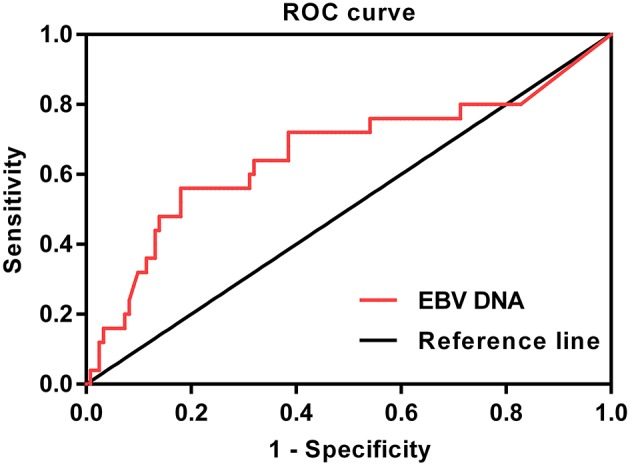
Receiver operating characteristic curve of pretreatment EBV DNA for DFS (AUC = 0.649).

In this study, ROC curve was used to evaluate different cutoff points for pretreatment plasma EBV DNA levels (Figure 1). The AUC of pretreatment EBV DNA for DFS was 0.649, with a sensitivity of 54.2% and a specificity of 81.3% using the cutoff value of 39,500. At this point, the Youden index (sensitivity + specificity−1) was considered to be maximal. In order to facilitate and promote the clinical application of this biomarker, 40,000 copies/mL was taken as the optimum cutoff value to classify the patients into low and high pretreatment EBV DNA groups for further statistical analysis.

The correlations between pretreatment plasma EBV DNA levels and various clinicopathological features were examined. There was no significant difference in age, gender, histology, body mass index (BMI), stage, chemotherapy, or total dose of cisplatin between patients with low and high plasma EBV DNA levels (all *P* > 0.05; Table 3).

**Table 3:** Baseline characteristics of the patients with children and adolescent NPC stratified by low vs. high pretreatment EBV DNA.

**Characteristics**	**EBV DNA**	**EBV DNA**	***P***
	**≤40,000**	**>40,000**	
	**(%, *n* = 113)**	**(%, *n* = 34)**	
Age, years			0.915
≤17	61 (54.0)	18 (52.9)	
>17	52 (46.0)	16 (47.1)	
Gender			0.109
Male	81 (71.7)	29 (85.3)	
Female	32 (28.3)	5 (14.7)	
Pathologic type			1.000^#^
WHO II	3 (2.7)	0 (0)	
WHO III	110 (97.3)	34 (100.0)	
Pretreatment BMI, kg/m^2^			1.000^*^
<23	98 (86.7)	30 (88.2)	
≥23	15 (13.3)	4 (11.8)	
T stage			0.603
T1	3 (2.7)	1 (2.9)	
T2	8 (7.1)	2 (5.9)	
T3	45 (39.8)	16 (47.1)	
T4	57 (50.4)	15 (44.1)	
N stage			0.564
N0	3 (2.7)	1 (2.9)	
N1	29 (25.7)	9 (26.5)	
N2	62 (54.9)	15 (44.1)	
N3	19 (16.8)	9 (26.5)	
Overall stage			0.748
I	1 (0.9)	0 (0)	
II	3 (2.7)	2 (5.9)	
III	44 (38.9)	11 (32.4)	
IV	65 (57.5)	21 (61.8)	
Combination with chemotherapy			0.092
No	2 (1.8)	1 (2.9)	
NAC	18 (15.9)	2 (5.9)	
CCT	28 (24.8)	6 (17.6)	
NAC + CCT	65 (57.5)	25 (73.5)	
Total dose of cisplatin, mg/m^2^ (mean ± SD)	275.8 ± 95.3	291.8 ± 105.3	0.403

# *Fisher exact test*.

* *Correction for continuity*.

*BMI, body mass index; NAC, neoadjuvant chemotherapy; CCT, concurrent chemotherapy; SD, standard deviation*.

### Survival Outcome

The median follow-up for the entire cohort was 58 months (range, 10–119 months), and the median failure times were 16 months (12–37 months) and 7 months (3–52 months) for local-regional recurrence and distant metastasis, respectively. The 5-year survival rates for all patients were as follows: OS, 88.7%; LRRFS, 95.2%; DMFS, 84.8%; and DFS, 81.5%. For patients receiving chemotherapy, the 5-year OS, LRRFS, DMFS, and DFS were 88.5, 95.1, 85.2, and 81.9%, respectively.

### Univariate and Multivariate Analyses

**Figure 2:**
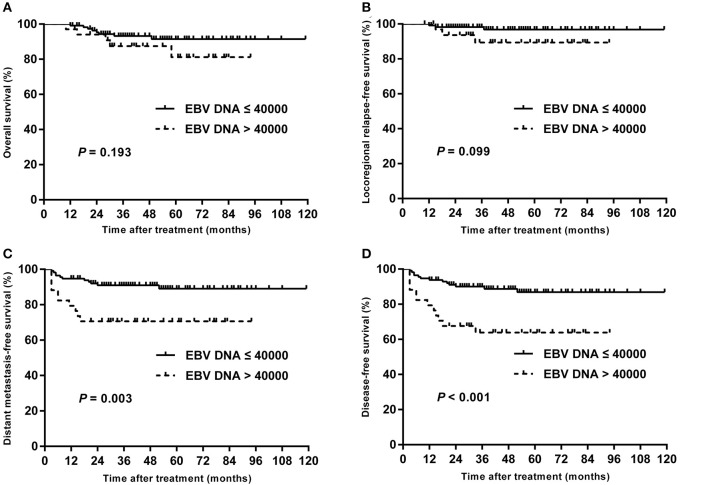
Kaplan–Meier estimate of the OS **(A)**, LRRFS **(B)**, DMFS **(C)**, and DFS **(D)** of children and adolescent NPC patients according to pretreatment EBV DNA levels.

Univariate analyses were performed using age, gender, BMI, T stage, N stage, chemotherapy, total dose of cisplatin and plasma EBV DNA levels as possible variables. As seen in Table 4, high plasma EBV DNA was significantly associated with poorer 5-year DMFS and DFS. The 5-year OS, LRRFS, DMFS, and DFS rates for high vs. low plasma EBV DNA group were 81.2 vs. 91.5% (*P* = 0.193), 89.4 vs. 96.8% (*P* = 0.099), 70.6 vs. 89.1% (*P* = 0.003), and 63.9 vs. 86.9% (*P* < 0.001), respectively (Table 4 and Figure 2). In addition, patients with advanced T stage had poorer 5-year OS, DMFS, and DFS. The Cox regression method was used, and the above factors were taken as covariates for analysis. The results revealed that high plasma EBV DNA was an independent predictor for DMFS and DFS, and T stage was significantly associated with OS, DMFS, and DFS (Table 5). Variables, including age, gender, BMI, T stage, N stage, total dose of cisplatin, and plasma EBV DNA levels, were also taken for multivariate analysis in 144 patients receiving chemotherapy, and the results were similar to those in the entire cohort (Supplemental Table 1).

**Table 4:** Univariate analysis of prognostic factors for 147 patients.

**Variate**	**5-Year survival rate (%)**							
	**OS**	***P***	**LRRFS**	***P***	**DMFS**	***P***	**DFS**	***P***
Age, years		0.927		0.809		0.878		0.823
≤17	88.5		96.8		84.6		81.9	
>17	88.7		94.3		84.9		80.9	
Gender		0.816		0.657		0.443		0.786
Male	89.2		95.8		83.4		81.2	
Female	87.6		93.4		89.0		82.7	
Pretreatment BMI, kg/m^2^		0.634		0.347		0.846		0.895
<23	88.3		94.5		85.0		81.3	
≥23	92.9		100		83.6		83.6	
T stage		0.008		0.317		0.002		<0.001
T1–T3	97.2		97.3		94.6		93.2	
T4	80.2		92.6		74.7		69.4	
N stage		0.629		0.829		0.585		0.540
N0–1	90.4		93.9		88.0		84.6	
N2–3	88.1		95.7		83.5		80.3	
Pretreatment EBV DNA, copies/mL		0.193		0.099		0.003		<0.001
≤40,000	91.5		96.8		89.1		86.9	
>40,000	81.2		89.4		70.6		63.9	
Combination with chemotherapy		0.632		0.751		0.252		0.318
No	100.0		100.0		66.7		66.7	
Yes	88.5		95.1		85.2		81.9	
Total dose of cisplatin, mg/m^2^		0.601		0.874		0.640		0.980
<320	87.8		94.6		85.2		80.3	
≥320	89.6		95.6		84.2		82.5	

*BMI, body mass index; OS, overall survival; LRRFS, locoregional relapse-free survival; DMFS, distant metastasis-free survival; DFS, disease-free survival*.

**Table 5:** Multivariate analysis of prognostic factors for 147 patients.

**Endpoint**	**Variate**	**HR**	**95% CI**	***P***
OS	T stage: T4 vs. T1–T3	6.62	1.45–30.15	0.015
	Pretreatment EBV DNA: >40,000 vs. ≤40,000 copies/mL	2.56	0.82–7.98	0.106
LRRFS	T stage: T4 vs. T1–T3	2.67	0.47–15.21	0.301
	Pretreatment EBV DNA: >40,000 vs. ≤40,000 copies/mL	4.63	0.84–25.37	0.078
DMFS	T stage: T4 vs. T1–T3	5.46	1.82–16.37	0.002
	Pretreatment EBV DNA: >40,000 vs. ≤40,000 copies/mL	3.86	1.61–9.23	0.002
DFS	T stage: T4 vs. T1–T3	5.20	1.94–13.91	0.001
	Pretreatment EBV DNA: >40,000 vs. ≤40,000 copies/mL	4.25	1.91–9.46	<0.001

*OS, overall survival; LRRFS, locoregional relapse-free survival; DMFS, distant metastasis-free survival; DFS, disease-free survival; HR, hazard ratio; CI, confidence interval*.

### Subgroup Analysis Stratified by T Stage

**Figure 3:**
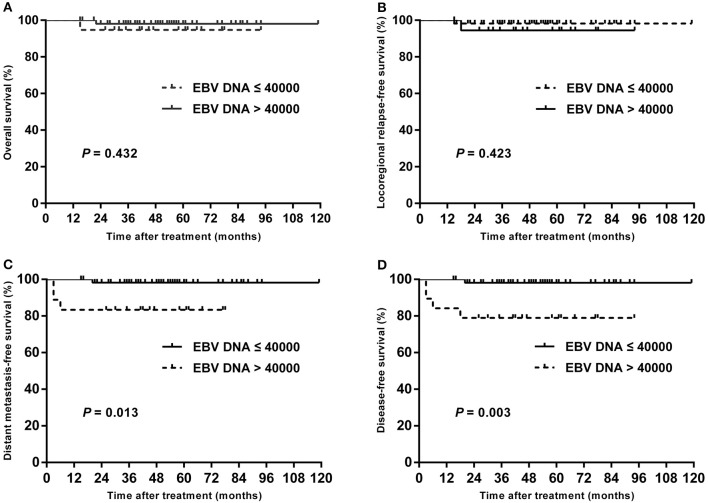
Kaplan–Meier estimate of the OS **(A)**, LRRFS **(B)**, DMFS **(C)**, and DFS **(D)** of T1–T3 children and adolescent NPC patients according to pretreatment EBV DNA levels.

**Figure 4:**
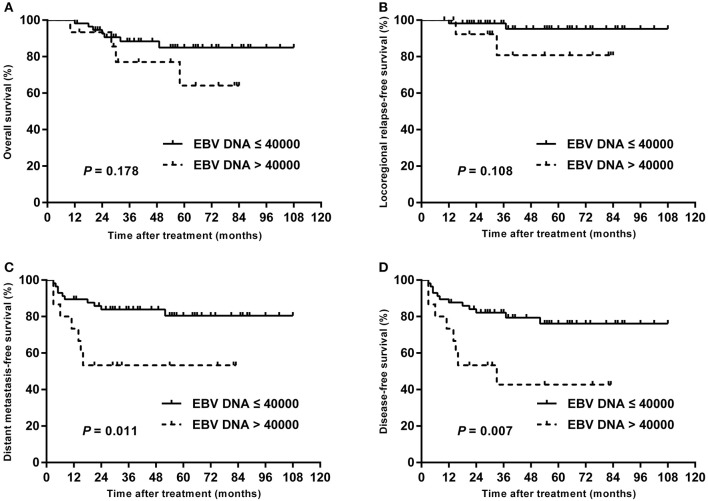
Kaplan–Meier estimate of the OS **(A)**, LRRFS **(B)**, DMFS **(C)**, and DFS **(D)** of T4 children and adolescent NPC patients according to pretreatment EBV DNA levels.

In subgroup analysis of T1–T3 disease, the patients with high plasma EBV DNA levels presented with worse DMFS (84.2 vs. 98.1%; *P* = 0.013) and DFS (78.9 vs. 98.1%; *P* = 0.003), but with similar OS (94.7 vs. 98.0%; *P* = 0.432) and LRRFS (94.4 vs. 98.2%; *P* = 0.423; Figure 3). For patients with T4, statistical significance was also achieved for DMFS (53.3 vs. 80.4%; *P* = 0.011) and DFS (42.7 vs. 76.1%; *P* = 0.007), but not for OS (64.2 vs. 85.0%; *P* = 0.178) and LRRFS (80.8 vs. 95.2%; *P* = 0.108; Figure 4).

## Discussion

Plasma EBV DNA has been reported to have prognostic value in patients with non-metastatic NPC treated with conventional RT or IMRT (14, 25, 26). The present study, which involved a large cohort of NPC patients in childhood and adolescence treated with IMRT, is the first one to provide valuable data on treatment outcomes and the clinical value of EBV DNA levels. We have shown that IMRT resulted in a favorable prognosis for children and adolescent NPC (5-year DFS, 81.5%), especially in patients with low pretreatment EBV DNA load (5-year DFS, 86.9%), which indicated that pretreatment EBV DNA level is an important prognostic factor in this age group.

In young patients, the predominant histology of NPC is an undifferentiated variant of disease (27–29), which was confirmed in our study where 98.0% of pediatric patients were histologically WHO type III. On the other hand, ~96.0% of our patients presented in advanced clinical stage III or IV, similar to other reports with stage III–IV patients accounting for 92.0–97.3% (5, 7, 29).

A previous study by Chou et al. (30) showed that NPCs are correlated with EBV DNA infection as the virus infects the epithelial cells promoting the activation of proliferation signaling. Studies also have demonstrated that the circulating cell-free EBV DNA is mainly released from apoptotic and necrotic cancer cells. Consequently, circulating cell-free DNA could reflect the tumor load of NPC patients (31). According to previous studies, pretreatment EBV DNA levels have a strong relation with clinical stages of NPC (14, 25, 26). In our study, although the patient with advanced-stage NPC had higher levels of pretreatment EBV DNA, a positive correlation was not found between pretreatment EBV DNA concentrations and T stage, N stage, and TNM stage grouping (all *P* > 0.05). One reasonable explanation for this negative correlation is that children with NPC differ from their adult counterparts in having a closer association with EBV. Therefore, some children with early-stage NPC may also have high levels of pretreatment EBV DNA. Additionally, pretreatment plasma EBV DNA may not precisely predict the tumor burden for patients with children and adolescent NPC as the circulating cell-free plasma EBV DNA load only originates from apoptotic and necrotic tumor cells, rather than all circulating tumor cells (32). Furthermore, the small sample size of patients in each group could potentially affect the results.

Interestingly, the cutoff value for pretreatment EBV DNA in this study was 40,000 copies/mL, which was much higher than that in previous studies. For example, Chan et al. (33) chose the cutoff value for pretreatment EBV DNA on the basis of a measure of heterogeneity with the log-rank test statistic and reported that a cutoff value of 4,000 copies/mL was optimal for classifying patients into two groups and demonstrated a highly statistically significant difference in progression-free survival. In the study by Lin et al. (14), the median concentration of EBV DNA (1,500 copies/mL) was chosen as cutoff value, and they found that OS (*P* < 0.001) and relapse-free survival (*P* = 0.02) were significantly lower among patients with pretreatment plasma EBV DNA concentrations of at least 1,500 copies/mL than among those with concentrations of <1,500 copies/mL. However, study subjects in these reports were almost adult patients. Considering that the patients in the present study were specifically children and adolescents with locoregionally advanced disease and a higher pretreatment EBV DNA level, previous cutoff points might not be suitable for our study. Therefore, the metrics used to describe the prognostic quality of each potential cutoff point in our study were the AUC, the sensitivity, and the specificity, which were calculated using ROC analysis. The sensitivity and specificity were simultaneously maximized in order to determine the optimal pretreatment EBV DNA cutoff points. The cutoff value identified in ROC curve analysis was 40,000 copies/mL, and there was an extreme difference in the DFS of patients with low vs. high pretreatment EBV DNA. Furthermore, the optimal cutoff point of the pretreatment EBV DNA (40,000 copies/mL) was confirmed to be an independent prognostic factor for DMFS and DFS in both entire cohort and patients receiving chemotherapy by multivariate analysis. A previous study by Shen et al. (15) also used ROC curve to determine the optimal cutoff value for pretreatment EBV DNA load in childhood and adolescent patients; however, they found that a cutoff level of 7,500 copies/mL could predict outcomes with the best trade-off between sensitivity and specificity. This lower cutoff point may be due to the fact that the patients in their study were older (median age, 19 years; range, 6–21 years). In addition, owing to the relatively short follow-up time in their study, the calculation of ROC curve was based on 3-year survival outcome, which might have an influence on evaluating the cutoff value. Generally, we would like to suggest that the pretreatment EBV DNA cutoff point should be set to 40,000 copies/mL for children and adolescent NPC patients treated with IMRT.

A higher T stage has been reported as an unfavorable factor for survival (27, 34). In the present study, higher plasma EBV DNA and T4 category were independent predictors for DMFS and DFS. Furthermore, higher plasma EBV DNA remained an independent unfavorable prognostic factor in subgroup analysis stratified by T category.

In the current study, IMRT has provided excellent locoregional control in children and adolescent NPC patients with LRRFS of 95.2%. Nevertheless, the NPC failure pattern was not altered by IMRT, and distant metastasis remained the major pattern of failure, a result similar to the findings of other reports (5, 35, 36). We observed that a level of pretreatment EBV DNA >40,000 copies/mL was highly statistically significantly associated with the poorer DMFS and DFS. Such a subgroup of patients may benefit from systemic treatment before RT, which can eradicate micrometastases earlier. The Children's Oncology Group ARAR0331 study showed excellent event-free survival (EFS) and OS of induction chemotherapy plus concurrent chemoradiotherapy (CCRT) in childhood NPC patients (37). However, a large population, retrospective study by Liu et al. (38) showed that in adult NPC patients the addition of neoadjuvant chemotherapy to CCRT could only reduce distant failure in patients with low risk of treatment failure (stage N0–1 disease and EBV DNA <4,000 copies/mL). In our study, 25 patients (73.5%) with high pretreatment EBV DNA received two to four cycles of induction cisplatin and 5-fluorouracil; or cisplatin and taxanes; or taxanes, cisplatin, and 5-fluorouracil, followed by cisplatin-based CCRT; however, the clinical outcome was far from satisfactory. On the other hand, previous studies by Ou et al. demonstrated that total dose of cisplatin more than 300 mg/m^2^ in the whole course of treatment indicates a favorable prognosis of DFS, DMFS, and OS in locally advanced NPC treated with IMRT (39, 40). ARAR0331 study also observed a trend toward increased EFS for patients assigned to receive higher doses of cisplatin during CCRT (37). In the present study, however, patients with high EBV DNA levels received similar cumulative cisplatin dose to those with low EBV DNA levels (*P* = 0.403). Therefore, future studies on the most effective regimens and ideal intensity of chemotherapy (e.g., cumulative cisplatin dose) in children and adolescent NPC patients with high EBV DNA levels are needed.

It should be noted that our study was subject to several limitations. First, the study is a retrospective series, which could have shortcomings such as selection bias. Second, because of the low incidence of childhood NPC, the number of patients who can be included is relatively limited, which might make the results of the study underpowered; thus, a larger sample size of patients is needed to confirm our findings. Third, the data were obtained exclusively at one center; therefore, these results must be validated by other datasets. The fourth concern was that we failed to include data regarding post-treatment EBV DNA; future studies need to continue to evaluate the prognostic value of post-treatment EBV DNA in children and adolescent NPC patients. Despite limitations above, this study still provides valuable reference for survival prediction of NPC patients in this age group.

## Conclusions

In this study, we described the long-term outcomes for patients with children and adolescent NPC treated with definitive IMRT. Our results suggest that pretreatment EBV DNA >40,000 copies/mL is an independent adverse prognostic factor on DMFS and DFS for this group of patients.

## Data Availability Statement

The datasets generated for this study are available on request to the corresponding author.

## Ethics Statement

The studies involving human participants were reviewed and approved by Human Ethics Approval Committee at Sun Yat-sen University Cancer Center. All subjects gave written informed consent in accordance with the Declaration of Helsinki.

## Author Contributions

WQ and YY conceived, designed and supervised the study. XL and XG collected and analyzed the data. WQ wrote the manuscript.

### Conflict of Interest

The authors declare that the research was conducted in the absence of any commercial or financial relationships that could be construed as a potential conflict of interest.
