# Supplementary material for: Clinical Implications of Plasma Epstein–Barr Virus DNA in Children and Adolescent Nasopharyngeal Carcinoma Patients Receiving Intensity-Modulated Radiotherapy
Source: Front Oncol. 2020 Mar 31;10:356. doi: 10.3389/fonc.2020.00356 (PMC7136458; doi:10.3389/fonc.2020.00356)
Supplement: Supplementary file 1 [file Table_1.DOCX]

**Supplemental Table 1 Multivariate analysis of prognostic factors for 144 patients receiving chemotherapy**

| Endpoint | Variate | HR | 95% CI | *P*-value |
| --- | --- | --- | --- | --- |
| OS | T stage: T4 vs. T1-3 | 6.37 | 1.40-29.02 | 0.017 |
|  | Pretreatment EBV DNA: >40000 copies/mL vs. ≤40000 copies/mL | 2.58 | 0.82-8.05 | 0.104 |
| LRRFS | T stage: T4 vs. T1-3 | 2.52 | 0.44-14.36 | 0.297 |
|  | Pretreatment EBV DNA: >40000 copies/mL vs. ≤40000 copies/mL | 4.67 | 0.85-25.71 | 0.077 |
| DMFS | T stage: T4 vs. T1-3 | 7.23 | 2.09-24.96 | 0.002 |
|  | Pretreatment EBV DNA: >40000 copies/mL vs. ≤40000 copies/mL | 3.53 | 1.44-8.66 | 0.006 |
| DFS | T stage: T4 vs. T1-3 | 6.37 | 2.16-18.74 | 0.001 |
|  | Pretreatment EBV DNA: >40000 copies/mL vs. ≤40000 copies/mL | 3.89 | 1.72-8.81 | 0.001 |

Abbreviations: OS, overall survival; LRRFS, locoregional relapse-free survival; DMFS, distant metastasis-free survival; DFS, disease-free survival; HR, hazard ratio; CI, confidence interval.
